# Blood pressure effects of SGLT2 inhibitors in kidney transplant recipients: a systematic review and meta-analysis

**DOI:** 10.1007/s11255-026-05162-9

**Published:** 2026-04-29

**Authors:** Abdullah Ahmad, Muhammad Hasnain Mankani, Abdullah Al-Kahil, Mahrukh Khan, Mariam Ismail, Mary Bilancini, Sapana Verma, Muhammad Ahmad Nadeem, Ahmed Sayed Ahmed

**Affiliations:** 1 Department of Medicine, Hamid Latif Teaching Hospital, Lahore, Pakistan; 2 The Aga Khan University, Karachi, Pakistan; 3 University Hospitals Parma Medical Center, Parma, OH USA; 4 Touro College of Osteopathic Medicine, Harlem, USA; 5 Transplantation Center, Department of General Surgery. Digestive Disease Institute, Cleveland Clinic, 2049 E 100Th Street, Cleveland, OH 44195 USA; 6 Bioinformatics Department, Northeastern University, Boston, MA USA

**Keywords:** Kidney transplantation, Hypertension, SGLT2 inhibitors, Blood pressure, Postoperative outcome, Systematic review, Meta-analysis

## Abstract

**Background:**

Post-transplant hypertension is common in kidney transplant recipients and contributes to cardiovascular risk and allograft dysfunction. Most available data come from studies where the primary indication for SGLT2 inhibitor use was post-transplant diabetes mellitus or cardiorenal protection, with blood pressure assessed as a secondary outcome.

**Objectives:**

To systematically evaluate the impact of SGLT2 inhibitors on blood pressure, metabolic and renal outcomes, and safety in kidney transplant recipients.

**Methods:**

PubMed, Embase, and Scopus clinical trial registries were systematically searched from inception to October 20, 2025. Randomized controlled trials and observational studies were included. Primary outcomes were changes in systolic and diastolic blood pressure at 3, 6, and 12 months. Secondary outcomes included body weight, glycated hemoglobin (HbA1c), renal function, and adverse events.

**Results:**

Twelve studies comprising 1,292 participants were included. In controlled difference-in-differences analyses (5 studies), SGLT2 inhibitors showed no significant blood pressure reductions versus control at any time point. Exploratory single-arm analyses suggested within-group systolic blood pressure reductions at 3 and 6 months; however, these estimates are at high risk of bias and cannot establish treatment effect.

**Conclusion:**

Exploratory single-arm analyses suggested modest short-term reductions in systolic blood pressure and suggested metabolic effects with an acceptable safety profile. However, controlled difference-in-differences analyses showed no significant blood pressure reductions versus control. Most available evidence derives from studies in which SGLT2 inhibitors were not initiated specifically for blood pressure control. Dedicated randomized controlled trials are required to determine their role in the management of post-transplant hypertension.

**Supplementary Information:**

The online version contains supplementary material available at https://doi.org/10.1007/s11255-026-05162-9.

## Introduction

Chronic kidney disease (CKD) affects more than 800 million individuals worldwide. It is one of the most prevalent and consequential non-communicable diseases globally. As CKD progresses, millions of patients ultimately develop end-stage renal disease (ESRD), requiring renal replacement therapy for survival [1]. Kidney transplantation remains the definitive treatment for ESRD, consistently demonstrating superior survival, improved quality of life, and substantially lower cardiovascular morbidity compared with long-term dialysis [2, 3]. Nevertheless, the long-term success of kidney transplantation continues to be challenged by the persistent risk of allograft dysfunction and failure.

A dominant mediator of this attrition is post-transplant hypertension, a condition affecting approximately 50–90% of kidney transplant recipients (KTRs) [4–6]. This is not merely a comorbid condition, but a major driver of adverse outcomes, with a strong and independent association with accelerated graft loss and increased cardiovascular mortality, the leading cause of death in this population [7–10]. The pathophysiology of post-transplant hypertension is largely iatrogenic; calcineurin inhibitors induce renal vasoconstriction and sodium retention, promoting a state of volume expansion. These effects are frequently compounded by corticosteroid exposure, persistent native kidney disease, and transplant renal artery stenosis [6, 11, 12].

Consequently, the management of post-transplant hypertension presents a critical therapeutic dilemma. Although clinical guidelines recommend several antihypertensive classes, their use is limited by significant side effects such as peripheral edema, hyperkalemia, and acute kidney injury that are particularly problematic in the fragile transplant milieu [13–16]. This highlights the need for alternative therapies that align with the unique pathophysiology of these patients [7, 15, 16].

Sodium–glucose cotransporter-2 (SGLT2) inhibitors have well-established cardiorenal benefits in broad patient populations [13, 17–19]. Their mechanism of action, which promotes natriuresis and osmotic diuresis, is uniquely suited to counteract the volume-expanded state central to post-transplant hypertension [20, 21]. Beyond blood pressure reduction, their potential to mitigate weight gain and improve glomerular hemodynamics confers a multifaceted therapeutic profile [17, 18, 20, 22].

Despite this biological rationale, the translation of SGLT2 inhibitors into the kidney transplant population remains uncertain [13, 19, 21, 23, 24]. The existing literature is fragmented, consisting of small, observational studies and underpowered trials that yield conflicting results on their efficacy and safety, particularly regarding infection risks in immunocompromised hosts [13, 17–21, 23–25]. This ambiguity has hindered their widespread adoption for hypertension management in KTRs [13, 19, 21, 23].

Therefore, to synthesize the available evidence regarding their blood pressure and metabolic effects, we conducted a systematic review and meta-analysis. This study aims to quantify the association of SGLT2 inhibitors with changes in systolic and diastolic blood pressure across multiple time points, recognizing that most included studies were not primarily designed to evaluate hypertension and assessed blood pressure as a secondary outcome. In these studies, SGLT2 inhibitors were initiated primarily for glycemic management of post-transplant diabetes mellitus or for cardiorenal protection. Our findings are intended to inform evidence-based clinical practice and guide the design of future definitive trials.

## Methods

### Protocol and registration

This review adhered to the methodological standards set by the Cochrane Handbook and followed the Preferred Reporting Items for Systematic Review and Meta-analysis (PRISMA) guidelines [26]. The protocol was registered with the International Prospective Register of Systematic Reviews (PROSPERO ID: CRD420251169311). This study used publicly available data and was exempt from institutional review board (IRB) approval.

#### Amendments to the protocol

In accordance with PRISMA 2020 recommendations (Item 24c), the authors report post-hoc amendments to the original study protocol. The initial protocol prioritized double-arm comparative analyses. However, during the screening phase, it was identified that high-quality comparative data for the primary hypertension outcomes were sparse. To maximize the synthesis of available evidence, the protocol was amended to include single-arm (pre–post) exploratory analyses for hypertension related outcomes only, which were analyzed separately from controlled comparisons to maintain methodological integrity. These analyses are strictly labeled as exploratory in this manuscript. Additionally, post hoc subgroup analyses were performed to further explore unexpected sources of statistical heterogeneity identified during the data synthesis.

### Eligibility criteria

Studies were included based on predefined criteria.

*Population*: Studies were selected based on predefined eligibility criteria. Eligible populations included adult (≥ 18 years) kidney transplant recipients with a functioning renal allograft. Studies enrolling both diabetic and non-diabetic transplant recipients were considered eligible.

*Intervention*: The intervention of interest was treatment with any SGLT2 inhibitor, including empagliflozin, dapagliflozin, canagliflozin, or ertugliflozin, at any dose and duration. In most included studies, SGLT2 inhibitors were initiated for indications such as post-transplant diabetes mellitus or cardiorenal protection rather than for the primary management of hypertension.

*Comparator*: For two-arm (controlled) studies: placebo, usual antihypertensive therapy, or no SGLT2 inhibitor use. For single-arm (pre-post) studies, the comparator was the baseline (pre-intervention) measurement. Trials with unclear comparator descriptions, overlapping treatment groups, or if comparisons are made with non-pharmacologic interventions (e.g., lifestyle modification only) were excluded.

*Outcomes*: The primary outcomes were the mean change in systolic blood pressure (SBP) and diastolic blood pressure (DBP) at 3-, 6-, and 12-month follow-up time points. Secondary outcomes included mean changes in body weight, glycated hemoglobin (HbA1c), serum creatinine, estimated glomerular filtration rate (eGFR), incidence of urinary tract infection (UTI), and incidence of hospitalizations due to any adverse event.

*Study design*: Randomized controlled trials (RCTs) and prospective or retrospective observational cohort studies were included. Case reports, case series with fewer than five participants, narrative or systematic reviews, editorials, commentaries, letters to the editor, animal studies, and conference abstracts were excluded.

### Search strategy and study selection

We conducted a systematic search of PubMed, Embase.com, and Scopus from inception to October 20, 2025, using a peer–reviewed search strategy. Additionally, we searched clinical trial registries (ClinicalTrials.gov and WHO International Clinical Trials Registry Platform) and gray literature sources (ProQuest Dissertations & Theses and Google Scholar). These searches identified no additional records beyond the database search. Only articles published in English were included, without any geographical limitations. Search strategy for each database can be found in Supplementary Table S1. The reference list of selected articles and text of appropriate reviews was manually screened to identify studies missed during the primary search.

All citations generated from the search were imported into the software Rayyan. Following duplicate removal, two reviewers (A.A. and M.H.M.) independently screened titles and abstracts for eligibility. Full-text articles of potentially relevant studies were then retrieved and assessed by the same two reviewers. Any disagreements regarding study inclusion were resolved by consensus or by a third senior reviewer (A.S.A.).

### Data extraction and quality assessment

Data extraction was performed independently by two reviewers (A.A. and M.I.) using a standardized data extraction form. Extracted data included study characteristics (first author, year of publication, study design, and sample size), baseline demographic and clinical characteristics of participants, and outcome data including means, standard deviations (SDs), and sample sizes for all reported outcomes at baseline and follow-up time points for both intervention and control groups, where applicable.

The risk of bias for each included study was assessed independently by two reviewers (A.A. and M.H.M.). The Cochrane Risk of Bias 2 (RoB 2) tool [27] was used for randomized controlled trials (RCTs), and the Risk of Bias in Non-randomized Studies of Interventions (ROBINS-I) tool [28] was used for observational studies. Considering the expected higher risk in the single-arm studies using ROBINS-I, these studies were also evaluated using the Joanna Briggs Institute (JBI) Critical Appraisal Checklist for Case Series to assess internal validity. Any disagreements regarding study inclusion were resolved by consensus or by consultation with a third senior reviewer (A.S.A.).

### Data synthesis and statistical analysis

Considering this review included both controlled and non-controlled studies, the analysis was stratified by study type. A random-effects model was used as the primary analytical approach for all meta-analyses to account for differences across studies [29, 30]. Statistical calculations were performed using Microsoft Excel, and data pooling was conducted using Review Manager (RevMan) version 5.4 and R (version 4) with the meta package [31–33].

#### Calculation of effect size

For all studies, the effect size was based on the change from baseline. The Mean_change_ was calculated using the following formula:

$${\mathrm{Mean}}_{{{\mathrm{change}}}} = {\mathrm{Mean}}_{{{\mathrm{final}}}} {-}{\mathrm{Mean}}_{{{\mathrm{baseline}}}} .$$

The standard deviation of the change (SD_change_) was calculated using:

$${\mathrm{SD}}_{{{\mathrm{change}}}} = \sqrt {\left[ {{\mathrm{SD}}_{{{\mathrm{baseline}}}}^{2} + {\mathrm{SD}}_{{{\mathrm{final}}}}^{2} - \left( {{2} \times Corr \times {\mathrm{SD}}_{{{\mathrm{baseline}}}} \times {\mathrm{SD}}_{{{\mathrm{final}}}} } \right)} \right]}$$

The standard error of the mean change (SE_change_) was then derived using:

$${\mathrm{SE}}_{{{\mathrm{change}}}} = {\mathrm{SD}}_{{{\mathrm{change}}}} /\sqrt {{\text{N where}}\;N\;{\text{represents the sample size}}} .$$

#### Single-arm (pre–post) study synthesis

For single-arm studies, the primary effect measure was the mean change from baseline for systolic blood pressure (SBP) and diastolic blood pressure (DBP) at relevant follow-up periods. Mean_change_ and SE_change_ were calculated for each study using the formulas described above and were pooled using the generic inverse variance (GIV) method in R via the metagen function. This analysis applied the Hartung–Knapp (HK) adjustment to generate more conservative confidence intervals.

#### Two-arm (controlled) study synthesis

For studies with a control arm, the effect size was calculated as the mean difference of change from baseline (MDCFB). This MDCFB estimate is mathematically equivalent to a difference-in-differences (DiD) estimate, reflecting the relative treatment effect between groups and representing (change in SGLT2i group) minus (change in control group). For all studies for each arm, the Mean_change_ derived. Then the following formula was used:

$${\mathrm{MDCFB}} = {\mathrm{Mean}}_{{{\mathrm{change}},{\mathrm{SGLT2i}}}} - {\mathrm{Mean}}_{{{\mathrm{change}},{\mathrm{control}}}} .$$

The standard error for MDCFB was calculated by combining the standard errors of both arms using:

$${\mathrm{SE}}_{{({\mathrm{MDCFB}})}} = \sqrt {\left[ {{\mathrm{SE}}_{{_{{{\mathrm{change}},{\text{ SGLT2i}}}} }}^{2} + {\mathrm{SE}}_{{{\mathrm{change}},{\text{ Control}}}}^{2} } \right]} .$$

When three or more studies reported results for an outcome, the pre-calculated MDCFB and SE_(MDCFB)_ values were pooled using the generic inverse variance method in RevMan to obtain pooled estimates.

#### Heterogeneity and sensitivity analysis

Statistical heterogeneity was quantified using the I^2^ statistic, with I^2^ values of < 40%, 40—70%, and > 70% considered low, moderate, and high, respectively. To further characterize the impact of extreme statistical heterogeneity (I^2^ > 90%), 95% prediction intervals (PI) were calculated for those specific outcomes.

Calculation of SD_change_ required correlation coefficient (Corr). If a study provided sufficient data, corr was calculated using the above mentioned formulas. In situations where this was not feasible, we imputed a standard correlation of Corr = 0.5 for our analysis.

To ensure robustness of results, sensitivity analysis was conducted by re-calculating and re-pooling the outcomes twice: once with a low correlation coefficient (Corr = 0.3) and once with a high correlation coefficient (Corr = 0.7). A "leave-one-out" sensitivity analysis was performed to identify the influence of individual outlying studies, in studies with high heterogeneity.

To address potential clinical heterogeneity, subgroup analyses were performed for blood pressure outcomes. Studies were stratified based on:

- glycemic status (diabetic, non-diabetic, or mixed cohorts);
- baseline hypertension (SBP > 140 mmHg vs. < 140 mmHg);
- post-transplant period (early/mixed vs. late > 1 year);
- stability of background antihypertensive therapy (stable regimen vs. adjusted during follow-up);
- blood pressure measurement methods (office vs. routine)

## Results

### Literature search synthesis

**Fig. 1:**
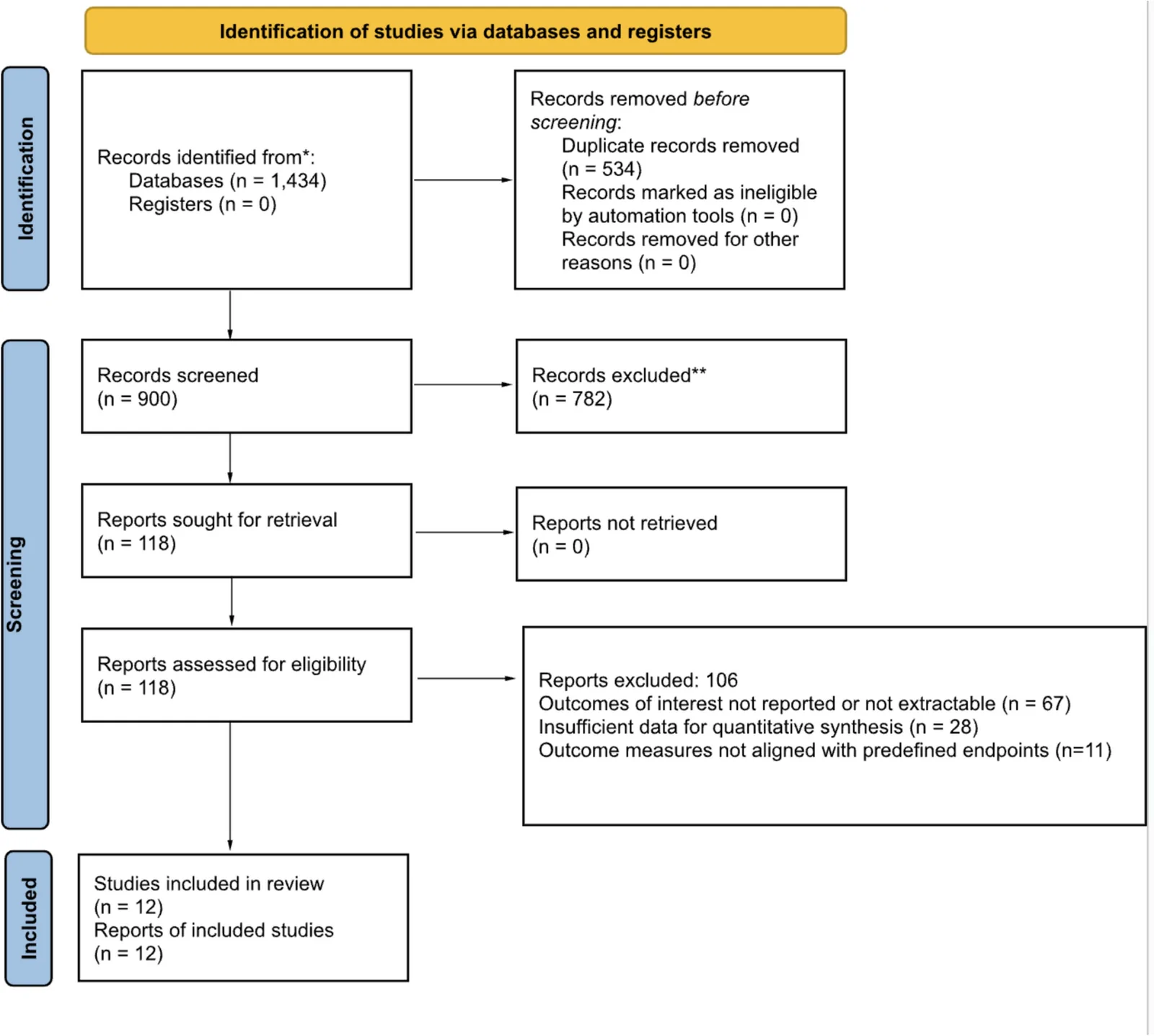
PRISMA flow diagram showing study selection process

The systematic search identified 1,434 records across PubMed, Embase, and Scopus. After removal of duplicates, 900 studies underwent title and abstract screening. Of these, 118 full-text articles were assessed for eligibility. Ultimately, 12 studies [34–42] met the inclusion criteria and were included in the qualitative and quantitative synthesis (Fig. 1).

### Baseline and study characteristics

This review utilized the data of 1,292 patients across 12 studies between 2018 and 2025. Seven studies [36–41, 44] were retrospective observational studies, four [35, 42, 43, 45] were prospective observational studies, and one study [34] was a prospective randomized controlled trial (RCT). Seven studies [35–37, 42–45] were single-arm observational cohorts, while five [34, 38–41] included a comparator group.

Treatment arms included various SGLT2 inhibitors (canagliflozin, empagliflozin, and dapagliflozin) with follow-up durations ranging from 6 to 12 months. Participants were predominantly older males, with mean ages between 49.1 and 63.0 years. Cardiometabolic risk factors were common, with mean HbA1c ranging from 6.5 to 9.3% and baseline systolic blood pressure between 123 and 150 mmHg.

Renal function was heterogeneous across cohorts, with baseline eGFR ranging from preserved function (75.8 mL/min/1.73 m^2^) to severe impairment (31 mL/min/1.73 m^2^). Study characteristics for all studies are reported in Table 1 and baseline characteristics of studies can be seen in Supplementary Tables S2 and S3. Across included studies, the primary indication for SGLT2 inhibitor use was predominantly post-transplant diabetes mellitus or cardiovascular risk reduction, with blood pressure outcomes typically reported as secondary endpoints.

**Table 1:** Study characteristics of individual studies

Study ID	Study design	Study setting	Specific SGLT2i administered	Longest follow-up available	Comparison type	Total patients	Control (if applicable)	BP method	BP timing	Antihypertensives
Halden 2019 (34)	Double-blind randomized controlled trial	Single center	Empagliflozin	6 months	Double arm	44	Placebo	Routine clinic BP (sitting)	Baseline + week 8 + week 16 + week 24	Mostly stable by protocol; reduced only if clinically indicated (2 patients reduced antihypertensives)
Hisadome 2021 (38)	Retrospective observational	Single center	Canagliflozin, ipragliflozin, luseogliflozin, empagliflozin, dapagliflozin, tofogliflozin	12 months	Double arm	89	Other oral hypoglycemic agents	Routine clinic BP (sitting)	Baseline + follow-up to 48 weeks (including intermediate serial assessments; final comparison at 48 weeks)	Mostly stable overall; minor change in α-blocker use only
Demir 2023 (41)	Retrospective observational	Single center	Not specified	12 months	Double arm	57	Other oral hypoglycemic agents	Not reported	Baseline + 3 months + 6 months + 12 months	Not Reported
Yeggalam 2023 (39)	Retrospective observational	Single center	Not specified	12 months	Double arm	114	Non-SGLT2i patients on Standard of Care treatment	Routine clinic BP (sitting)	Baseline + 3 months + 6 months + 12 months	Adjusted/changed during follow-up
Mahmoud 2023 (40)	Retrospective observational	Single center	Canagliflozin	12 months	Double arm	168	Non-SGLT2i patients on standard of care treatment	Routine clinic BP (sitting)	Baseline + 1 month + 3 months + 6 months + 12 months	Adjusted/changed during follow-up
Bilancini 2025 (45)	Prospective observational	Single center	Dapagliflozin	6 months	Single arm	66	Not applicable	Routine clinic BP (sitting)	Baseline + 3 months + 6 months	Stable
Maigret 2024 (43)	Prospective observational	Multicenter (13 centers)	Not Specified	6 months	Single arm	347	Not applicable	Routine clinic BP (sitting)	Baseline + 3 months + 6 months	Adjusted/changed during follow-up
Fructoso 2023 (42)	Prospective observational	Multicenter (18 centers)	Canagliflozin, Empagliflozin, Dapagliflozin, Ertugliflozin	12 months	Single arm	339	Not applicable	Routine clinic BP (sitting)	Baseline + 6 months (+ 12 months when available)	Adjusted/changed during follow-up
Shah 2019 (37)	Retrospective observational	Single center	Canagliflozin	6 months	Single arm	24	Not applicable	Routine clinic BP (sitting)	Baseline + serial follow-up visits up to 6 months (BP checked each visit)	Adjusted/changed during follow-up
Schwaiger 2018 (35)	Prospective observational	Single center	Empagliflozin	12 months	Single arm	14	Not applicable	Routine clinic BP (sitting)	Baseline + 2 weeks + 4 weeks + 3 months + 6 months + 12 months	Adjusted/changed during follow-up
Quilis 2024 (44)	Retrospective observational	Single center	Not Specified	6 months	Single arm	22	Not applicable	Routine clinic BP (sitting)	Baseline + 6 months	Stable
AlKindi 2019 (36)	Retrospective observational	Single center	Not Specified	12 months	Single arm	8	Not applicable	Routine clinic BP (sitting)	Baseline + 3 months + 6 months + 9 months + 12 months	Adjusted/changed during follow-up

### Quality assessment

Halden et al., the only RCT included in this review, was judged to have a low risk of bias across all domains using the Cochrane Risk of Bias 2 (RoB 2) tool [27]. Using the Cochrane ROBINS-I tool, all 11 observational studies were assessed as having a serious risk of bias (Supplementary Figures S1 and S2). Nine studies [36–44] exhibited serious risk in Domain 1 ("Bias due to confounding"). Serious risk was also identified in Domain 2 ("Bias in selection of participants into the study") and Domain 5 ("Bias due to missing data").

The seven single-arm studies [35–37, 42–45] were further evaluated using the Joanna Briggs Institute (JBI) Critical Appraisal Checklist for Case Series (Supplementary Table S4). Three studies were rated high quality [42–44], one moderate [35], and three low quality [36, 37, 45]. Detailed quality assessment explanations are provided in the Supplementary File. The overall certainty of evidence for key outcomes was low to very low according to GRADE assessment (Supplementary Table S5).

### Primary outcome: efficacy in blood pressure control

Where available, difference-in-differences (DiD) estimates (change in SGLT2i group minus change in control group) were prioritized for inference, while single-arm analyses were interpreted as supportive and exploratory.

#### Comparative analysis: SGLT2 inhibitors vs control/placebo

Data from Yeggalam et al. [39], showed no significant differences in mean SBP or DBP change from baseline between SGLT2 inhibitors and control arms at 3 months (*p* = 0.98 and *p* = 0.79, respectively). This finding of non-significance remained consistent at both 6 months (SBP: *p* = 0.29; DBP: *p* = 0.43) and 12 months (SBP: *p* = 0.65; DBP: *p* = 0.43).

At 12 months, Hisadome et al. reported a significant mean difference in SBP change from baseline (*p* = 0.045), while DBP change was not significant (*p* = 0.68) [38]. Mahmoud et al. reported no differences in mean SBP (*p* = 0.21) or DBP (*p* = 0.43) at 12 months [40]. Among placebo-controlled RCTs, Halden et al. found no significant differences in SBP or DBP change at 6 months [34]. Refer to Table 2 for study-level mean differences between SGLT2 inhibitors and comparator arms.

**Table 2:** Summary of pooled mean differences in systolic and diastolic blood pressure at 3, 6, and 12 months

Time point	Outcome	No. of studies	Mean difference (MD, mmHg)	95% CI	*p*-value	*I*^2^ (%)
3 months	SBP	4 (36,39,43,45)	− 5.66	− 6.86 to − 4.45	*p* < 0.001	0%
6 months	SBP	9 (34–37, 39,42–45)	− 4.80	− 7.24 to − 2.37	*p* < 0.001	29.9%
12 months	SBP	5 (35–36, 38–39, 42)	− 2.73	− 9.99 to + 4.53	*p* = 0.35	76.4%
3 months	DBP	3 (36,39,45)	− 2.67	− 6.30 to 0.96	*p* = 0.087	0%
6 months	DBP	8 (34–37,39, 42–43, 45)	+ 0.97	− 12.39 to + 14.33	*p* = 0.87	96.2%
6 months (excluding AlKindi et al.)	DBP	7 (34–35,37,39, 42–43, 45)	− 3.41	− 6.59 to − 0.23	*p* = 0.04	68.1%
12 months	DBP	5 (35–36, 38–39, 42)	− 3.11	− 7.17 to + 0.95	*p* = 0.10	44.6%

#### Single-arm analysis: pooled change in blood pressure

For SBP, pooled analysis suggested a reduction at 3 and 6 months. However, the pooled effect was not significant at 12 months. To address potential bias from SD imputation, empirical correlation coefficients (Corr) were derived from studies providing complete variance data. At 6 months (Fructoso et al.), calculated Corr values were 0.46 (SBP) and 0.52 (DBP) [42]. At 12 months, data from Hisadome et al. yielded a Corr of 0.50 for DBP; however, the SBP data from the same study resulted in a negative correlation (Corr = −0.14) [38]. Since a negative correlation is clinically implausible for blood pressure tracking, this value was excluded as a valid estimate after discussion among the authors.

**Fig. 2:**
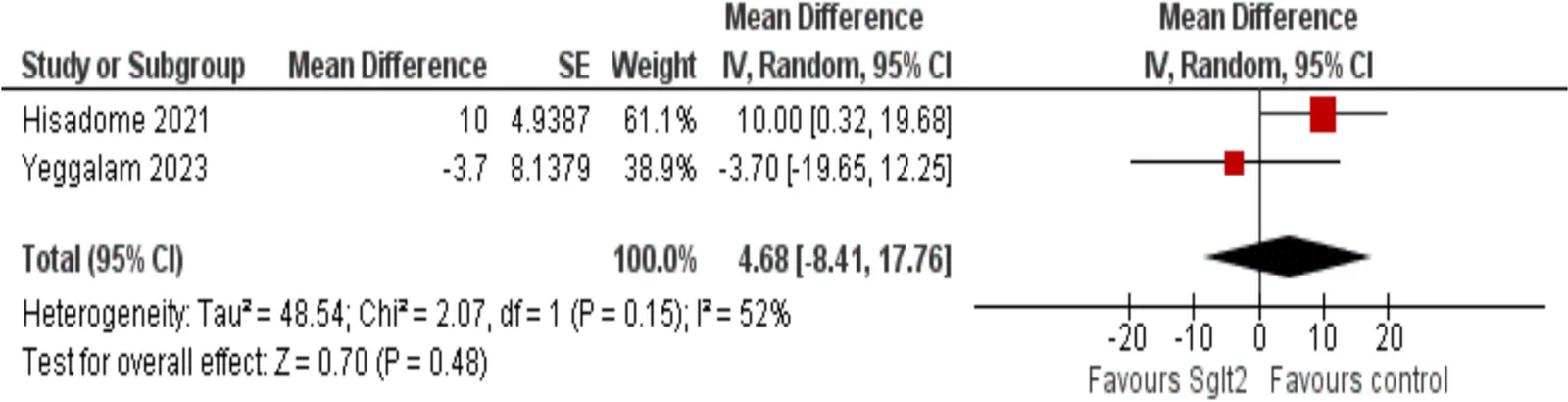
Forest plot of pooled change in systolic blood pressure at 3, 6, and 12 months in single-arm studies

As empirically derived correlation coefficients consistently clustered around 0.5, this value was selected as a conservative and representative estimate for the primary single-arm analysis across all time points. Sensitivity analyses, conducted by varying the imputed correlation coefficient (0.3 and 0.7), confirmed the robustness and significance of the 3-month and 6-month SBP findings as shown in Supplement Table S6. Forest plots for pooled changes in systolic blood pressure across follow-up time points are shown in Fig. 2.

For DBP, the primary analysis found no significant pooled reduction in DBP at any time point. One study (AlKindi et al.) reported an extreme DBP change (+ 41.87 mmHg), likely due to a typographical error; excluding this study reduced heterogeneity (I^2^) from 96.2% to 68.1% (95% PI: −20.85 to 22.80 mmHg). After sensitivity analysis by excluding this study, the pooled effect size became significant, the heterogeneity decreased (I^2^ = 68.1%) and the PI narrowed (−9.30 to 2.48). Refer to Table 3 for a summary of the pooled mean difference of SBP and DBP at all three follow-up points. Forest plots for systolic blood pressure at individual follow-up time points and corresponding diastolic blood pressure analyses, including sensitivity analyses, are provided in Supplementary Figures S3–S10.

**Table 3:** Calculated mean difference in change from baseline for individual outcomes at all follow-ups for each study

	Yeggalam 2023		Hisadome 2021	Demir 2023	Halden 2019	Mahmoud 2023
	Mean difference (95% CI)		Mean difference (95% CI)	Mean difference (95%CI)	*p*-value	*p*-value
SBP, mmHg						
3 months		0.20 (−12.74 to 13.14)	Not available	Not available	Not available	Not available
6 months		−6.30 (−18.16 to 5.56)	Not available	Not available	*p* = 1	Not available
12 months		−3.70 [−19.65, 12.25]	10.00 [0.32 to 19.68]	Not available	Not available	*p* = 0.21
DBP, mmHg						
3 months		1.10 (−7.11 to 9.31)	Not available	Not available	Not available	Not available
6 months		−2.80 (−9.79 to 4.19)	Not available	Not available	*p* = 1	Not available
12 months		−2.20 [−10.12 to 5.72]	−1.00 [−5.68 to 3.68]	Not available	Not available	*p* = 0.43
Body weight, kg						
3 months		−3.60 (−14.47 to 7.27)	Not available	Not available	Not available	Not available
6 months		−4.50 (−15.40 to 6.40)	Not available	Not available	*p* = 0.104	Not available
12 months		−4.90 [−9.59, −0.21]*	−2.30 [−4.53, −0.07]*	Not available	Not available	*p* = 0.01^*^
HbA1c, %						
3 months		−0.00 (−1.05 to 1.05)	Not available	−2.00 (−3.22 to −0.78)*	Not available	Not available
6 months		0.10 [−0.96, 1.16]	Not available	−1.73 [−2.52 to −0.94]*	Not available	Not available
12 months		−0.70 [−1.14, −0.26]*	0.10 [−0.34, 0.54]	−2.12 [−2.84, −1.40]*	Not available	*p* = 0.002*
eGFR, mL/min/1.73 m^2^						
3 months		−5.50 (−15.67 to 4.67)	Not available	1.77 (−8.67 to 12.21)	Not available	Not available
6 months		0.70 [–9.69, 11.09]	Not applicable	7.53 [−2.77, 17.83]	p = 1	Not applicable
12 months		1.80 [–4.40, 8.00]	2.10 [−1.14, 5.34]	8.86 [4.36, 13.36]*	Not available	p = 0.025
Creatinine, mg/dL						
3 months		0.10 (−0.14 to 0.34)	Not available	Not available	Not available	Not available
6 months		1. (−0.29 to 0.29)	Not available	Not available	Not available	Not available
12 months		−0.20 [−0.41, 0.01]	Not available	−0.05 [−0.23, 0.13]	Not available	Not available

^*^Indicates a statistically significant mean difference (*p* < 0.05)

Subgroup analyses were performed across all six outcomes to evaluate the impact of diabetes status, medication stability, baseline hypertension, blood pressure measurement method, and transplant vintage (Supplementary Table S7). For SBP at 6 months, a significant subgroup difference was observed regarding the post-transplant period (p = 0.017), with a more pronounced reduction in early or mixed cohorts (MD: −7.75 mmHg; 95% CI −12.00, −3.50) compared to late-transplant patients (MD: −4.57 mmHg; 95% CI −7.72 to −1.42). At 12 months, SBP reduction was significantly influenced by medication stability (p < 0.0001); cohorts with adjusted antihypertensive regimens demonstrated a consistent reduction (MD: −5.14 mmHg; 95% CI −6.82 to −3.46), whereas the single study with a stable regimen showed a paradoxical increase (MD: 6.00 mmHg; 95% CI 0.81–11.19). For DBP, a significant subgroup interaction occurred at 12 months regarding baseline hypertension (p = 0.024), where patients with high baseline blood pressure experienced a substantially larger reduction (MD: −10.00 mmHg; 95% CI −16.69 to −3.31) than those with normal baseline levels (MD: −2.21 mmHg; 95% CI −3.93 to −0.49). Notably, no significant differences were observed across any time point based on diabetes status or blood pressure measurement method (*p* > 0.05).

### Secondary outcomes

#### Metabolic outcomes

Yeggalam et al. demonstrated a significant difference in body weight change at 12 months between SGLT2 inhibitors and control groups (*p* = 0.041), but not at 3 or 6 months [39]. Hisadome et al. similarly reported a significant 12-month weight reduction (*p* = 0.043) [38]. Mahmoud et al. initially observed a 12-month weight difference (*p* = 0.01), which became non-significant after adjustment for multiple comparisons (*p* = 0.701) (40). Halden et al. reported a significant 6-month median weight change (*p* = 0.014) [34]. Additional pooled analyses for HbA1c at earlier time points and for body weight are shown in Supplementary Figures S11–S12.

**Fig. 3:**
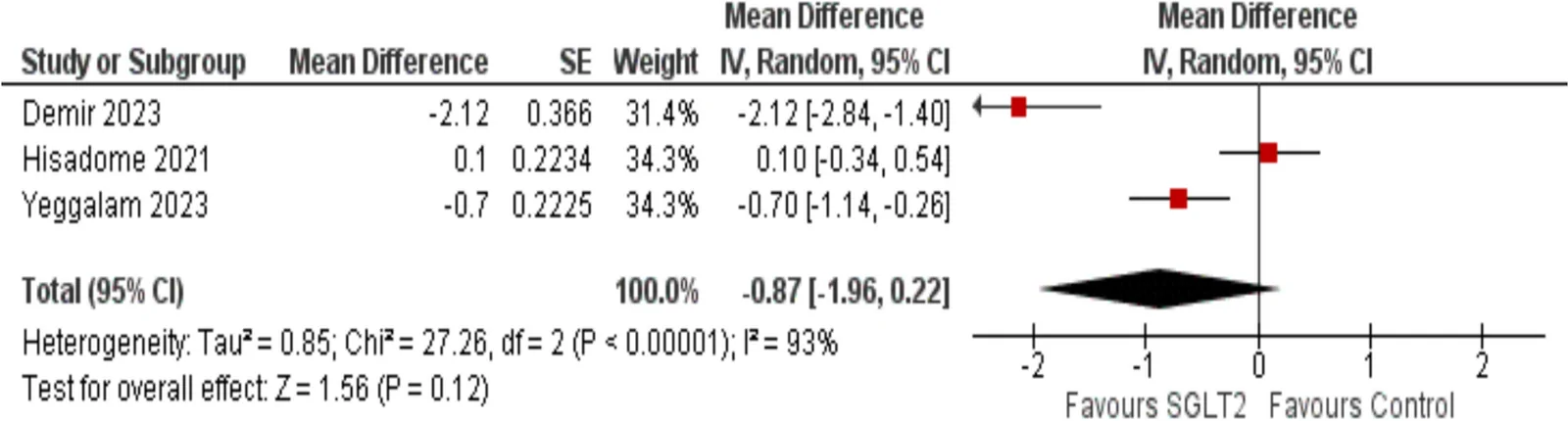
Forest plot comparing change in systolic blood pressure between SGLT2 inhibitor and control groups at 12 months

Regarding HbA1c, our analysis showed a significant difference in the mean change from baseline at 3 months for Demir et al. (*p* = 0.02), while Yeggalam et al. showed a non-significant result (*p* = 1.0) [39, 41]. This pattern was consistent at 6 months (Demir et al.: *p* < 0.001; Yeggalam et al.: *p* = 0.85) [39, 41]. For the 12-month time point, the mean differences from three studies were pooled. The overall pooled effect size remained non-significant between the SGLT2 inhibitors and control groups (MD: −0.87; 95% CI −1.96 to 0.22; *p* = 0.12; *I*^2^ = 93%; Fig. 3) and the 95% CI was wide (−12.28 to + 10.54%).

Consistent with their body weight findings, Mahmoud et al. reported that an initial difference in the median change in HbA1c at 12 months became non-significant after adjusting for multiple comparisons [40]. Halden et al. reported a significant difference in the median change from baseline at 6 months between the SGLT2 inhibitors and placebo groups [34].

### Renal outcomes

**Fig. 4:**
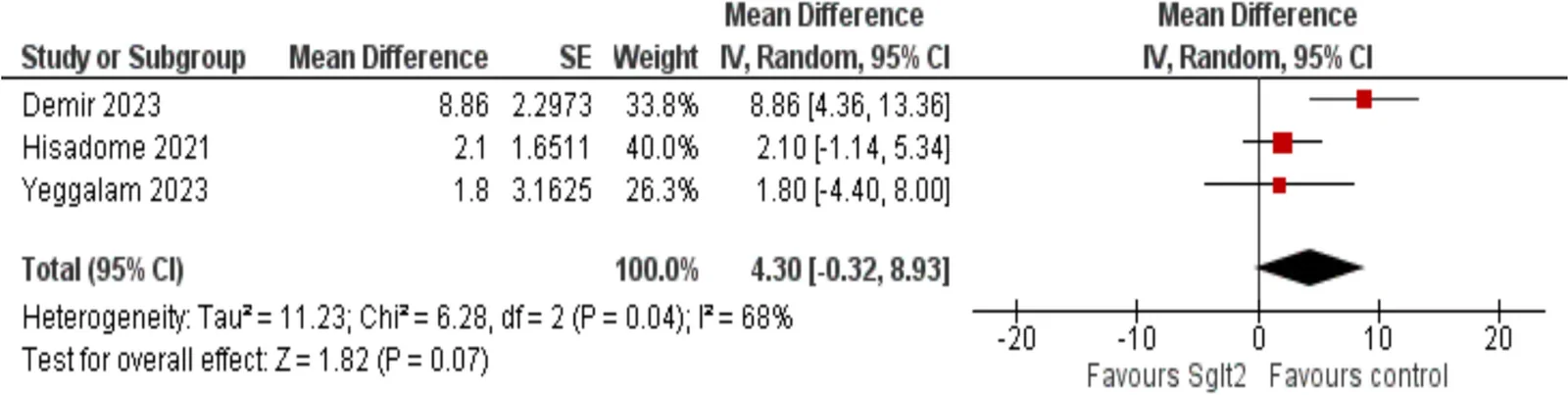
Forest plot of pooled change in estimated glomerular filtration rate at 12 months

Analysis of data from Yeggalam et al. and Demir et al. showed no difference in the mean change of eGFR from baseline at 3 or 6 months (Yeggalam: *p* = 0.28 and 0.90; Demir: *p* = 0.74 and 0.16) [39, 41]. For the 12-month time point, a pooled analysis of three studies was conducted, which also showed no significant difference in the change in eGFR from baseline (MD: 4.30; 95% CI −0.32 to 8.93; *p* = 0.07; *I*^2^ = 68%; Fig. 4).

In contrast, Mahmoud et al. reported an improvement in eGFR over 12 months in the SGLT2 inhibitors group compared to the control group (*p* = 0.025) [40]. Halden et al. reported no difference in the median change of eGFR from baseline at 6 months between the SGLT2 inhibitors and placebo groups [34].

Regarding the change in serum creatinine from baseline, analysis of data from Yeggalam et al. found no significant difference between the SGLT2 inhibitors and control groups at 3, 6, or 12 months (*p* = 0.41, *p* = 1.0, and *p* = 0.06, respectively) [39]. Demir et al. showed similar results, with no significant difference found at 12 months between the SGLT2 inhibitors and control groups (*p* = 0.62) [41]. Additional renal outcome analyses are shown in Supplementary Figures S13–S15.

#### Safety outcomes

**Fig. 5:**
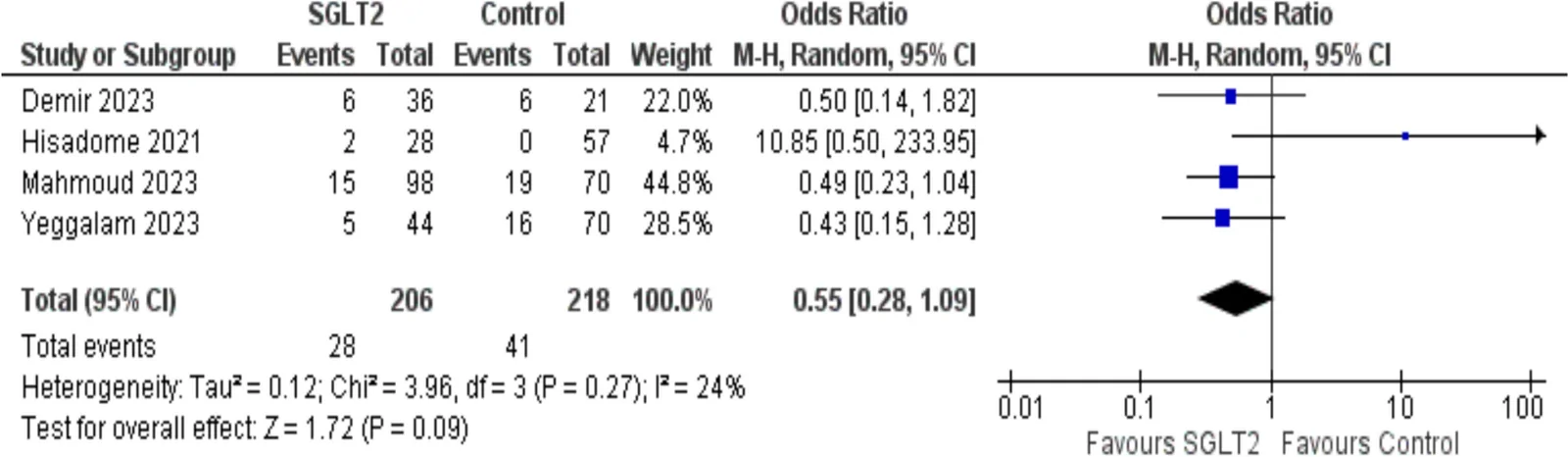
Odds ratio for urinary tract infection comparing SGLT2 inhibitors versus control

To assess the effect of SGLT2i on the incidence of UTIs, data from four studies were pooled. This analysis revealed that the odds of UTI in the SGLT2 inhibitors group were not significantly different compared to the control group (OR: 0.55; 95% CI 0.28–1.09; *p* = 0.09; *I*^2^ = 24%; Fig. 5).

**Fig. 6:**
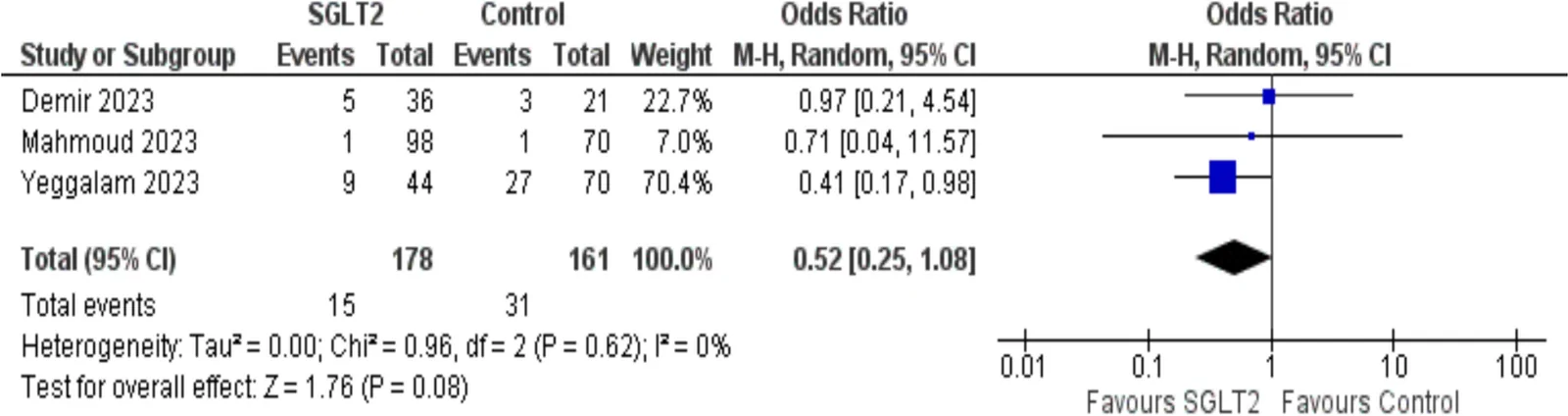
Odds ratio for hospitalization due to adverse events

Similarly, no significant difference was found in the odds of hospitalization due to any adverse events between the SGLT2 inhibitors and control groups (*n* = 4 studies; OR: 0.52; 95% CI 0.25–1.08; *p* = 0.08; *I*^2^ = 0%; Fig. 6).

Other safety endpoints were reported inconsistently across studies. Genital mycotic infections were mentioned in two studies, with no clear increase in SGLT2i groups. No cases of diabetic ketoacidosis (including euglycemic DKA) were reported in any study. Acute kidney injury was not systematically reported, but no study identified a safety signal. Rejection episodes were reported in three studies; Demir et al. noted lower rejection rates in the SGLT2i group, while others found no difference. Tacrolimus and cyclosporine trough levels were assessed in two studies (Halden et al., Hisadome et al.), both confirming stable concentration-to-dose ratios during SGLT2i therapy.

## Discussion

This systematic review and meta-analysis synthesizes available evidence on blood pressure effects of SGLT2 inhibitors in kidney transplant recipients. Our findings demonstrate modest short-term reductions in systolic blood pressure, suggested metabolic effects, and an acceptable safety profile.

### Blood pressure effects and clinical relevance

In evaluating the impact of SGLT2 inhibitors on post-transplant hypertension, our primary inference is drawn from controlled comparative analyses. In these head-to-head comparisons, SGLT2 inhibitors generally did not demonstrate a statistically significant difference-in-differences (the mean difference of change from baseline between SGLT2i and control groups) for SBP or DBP at most time points.

While nominal SBP reductions were observed in pooled single-arm (pre-post) analyses at 3 and 6 months, these findings are strictly exploratory. Since single-arm changes cannot isolate a true treatment effect from natural variation, regression to the mean, or background therapy adjustments, they must be interpreted with significant caution. These nominal findings align with meta-analyses in non-transplant populations demonstrating consistent blood pressure lowering effects of 3–5 mmHg [46, 47], and the modest reductions seen in the EMPA-KIDNEY trial [48]. However, without a consistent significant difference in our controlled comparisons, these single-arm drops cannot be definitively attributed to the SGLT2 inhibitor alone.

The discrepancy between the significant SBP drops seen in treated single-arm cohorts at 3 and 6 months and the non-significant comparative results at 12 months likely reflects high clinical variability. Our random-effects models, which prioritize a conservative account of study-level heterogeneity, suggest that the blood pressure–lowering effect may not be as robust as initial observational data suggested. Subgroup analyses suggest that these effects may be transient or highly dependent on clinical context, as SBP reductions were significantly more pronounced in early transplant cohorts and heavily influenced by background anti-hypertensive medication adjustments.

Diastolic blood pressure (DBP) reductions were notably more modest and less consistent in the single-arm analysis. The primary 6-month DBP analysis was characterized by extreme statistical heterogeneity and a wide 95% prediction interval, indicating that the treatment effect is variable across different clinical settings. This unpredictability is further evidenced by subgroup data showing that 12-month DBP reductions were significantly larger only in patients with high baseline hypertension. This pattern hints toward a possible preferential effect of SGLT2 inhibitors on systolic rather than diastolic blood pressure, likely mediated by natriuresis and reductions in arterial stiffness rather than direct vasodilation [49]. Such predominantly systolic benefits may be particularly relevant in transplant recipients, who frequently exhibit increased arterial stiffness related to chronic kidney disease and long-term immunosuppressive therapy [50]. While even small SBP reductions (e.g., 2 mmHg) are associated with a 7% reduction in cardiovascular mortality in the general population [51], this review underscores that definitive evidence for a superior blood pressure-lowering effect over standard care in the transplant setting is currently lacking.

### Metabolic benefits in post-transplant care

The observed reductions in body weight address a critical therapeutic gap in post-transplant metabolic management. Post-transplant weight gain affects up to 80% of kidney transplant recipients and is strongly associated with new-onset diabetes, metabolic syndrome, and cardiovascular disease [52, 53]. Our pooled analyses demonstrated that SGLT2 inhibitor therapy was associated with statistically significant and consistent reductions in body weight at 12 months, with minimal heterogeneity across studies. These findings align with well-established mechanisms of SGLT2 inhibitors, including glucosuria-induced caloric loss and favorable effects on body composition through preferential reduction of visceral adipose tissue [54, 55].

Improvements in glycemic control, as reflected by HbA1c, revealed a more variable pattern. While several individual studies demonstrated reductions in HbA1c, pooled estimates at 12 months did not consistently reach statistical significance, particularly in the presence of substantial heterogeneity. The wide 95% prediction interval (PI), further suggests that the glycemic response is highly unpredictable across different cohorts and should be viewed as exploratory. This variability may reflect differences in baseline glycemic status, concomitant diabetes medications, and varying degrees of steroid-induced hyperglycemia across studies. Nevertheless, the overall directionality of effect consistently favored SGLT2 inhibitors, suggesting a clinically meaningful trend in a population at elevated risk for post-transplant diabetes and metabolic complications. Post-transplant diabetes and inadequate glycemic control are well-recognized determinants of both graft survival and patient outcomes [56, 57].

Importantly, the potential benefits of SGLT2 inhibitors may extend beyond glucose lowering alone. The DAPA-CKD trial demonstrated that dapagliflozin reduced kidney function decline and cardiovascular events independent of baseline glycemic status [58, 59], supporting pleiotropic effects. In the transplant setting, where corticosteroids and calcineurin inhibitors contribute substantially to insulin resistance and hyperglycemia, SGLT2 inhibitors offer an insulin-independent metabolic strategy that circumvents these iatrogenic challenges [60].

### Renal safety and allograft preservation

The stability of renal function parameters addresses fundamental concerns regarding SGLT2 inhibitor effects on allograft function. Our analysis showed no significant differences in eGFR or serum creatinine changes between SGLT2 inhibitor and control groups at 12 months, supporting a reassuring renal safety profile. These findings are consistent with mechanistic data suggesting SGLT2 inhibitors exert nephroprotective effects through reductions in hyperfiltration injury, decreased tubular workload, and improved renal oxygenation [61, 62].

The initial eGFR decline reported with SGLT2 inhibitor initiation in non-transplant populations reflects hemodynamic adaptation mediated by reduced intraglomerular pressure, rather than structural kidney injury [63, 64]. Large randomized trials such as EMPA-KIDNEY demonstrated 28% reduction in kidney disease progression with empagliflozin [48], while DAPA-CKD showed sustained long-term nephroprotective benefits [58]. The absence of significant eGFR decline in our pooled analysis, coupled with overall stability over 12 months, is encouraging given the progressive nature of chronic allograft dysfunction.

The observation of improved eGFR in some individual studies, though not consistently replicated in pooled analyses, raises the possibility of longer-term nephroprotective effects warranting further evaluation in adequately powered trials with extended follow-up.

### Safety profile in immunocompromised hosts

The safety analysis provides crucial reassurance regarding infection risk in immunosuppressed transplant recipients. The pooled analysis of urinary tract infections showed no significant increase with SGLT2 inhibitors compared to controls. While SGLT2 inhibitors associate with genital mycotic infections in non-transplant populations [65, 66], serious urinary tract infection risk appears minimal in this cohort, consistent with findings from the CANVAS program and DECLARE-TIMI 58 trial [67, 68].

Despite theoretical concerns that immunosuppression heightens infection susceptibility, our findings suggest SGLT2 inhibitors do not meaningfully exacerbate this risk. This may reflect the predominantly local rather than systemic nature of SGLT2 inhibitor-associated infections and the effectiveness of contemporary prophylaxis protocols [68]. Crucially, the evidence indicates a lack of significant pharmacological interference. Halden et al. and Hisadome et al. both confirmed that trough levels and concentration-to-dose ratios for tacrolimus and cyclosporine remained stable during SGLT2i therapy [34, 38].

The absence of increased hospitalizations due to adverse events further supports overall tolerability, particularly relevant given transplant recipients’ heightened vulnerability to medication-related adverse effects [69].This is further supported by the lack of increased acute rejection, with Demir et al. even noting lower rejection rates in the SGLT2i group [41]. Genital mycotic infections were not consistently reported across studies; where reported, incidence appeared low, though underreporting cannot be excluded. No cases of diabetic ketoacidosis or euglycemic ketoacidosis were reported in the included studies. Acute kidney injury and volume depletion were not systematically reported but no signal was detected in individual studies.

### Mechanistic rationale and clinical context

The biological plausibility of SGLT2 inhibitor efficacy in post-transplant hypertension is supported by their mechanism of action. Calcineurin inhibitors, the cornerstone of immunosuppression, induce sodium retention, renal vasoconstriction, and sympathetic activation [70, 71]. SGLT2 inhibitors directly counteract these effects through natriuresis, osmotic diuresis with volume contraction, reduced arterial stiffness, and improved endothelial function [72, 73]. Additionally, SGLT2 inhibitors modulate tubuloglomerular feedback through restored sodium delivery to the macula densa, reducing intraglomerular pressure [74]. This may particularly benefit transplanted kidneys exhibiting early hyperfiltration, a phenomenon associated with accelerated chronic injury [75].

The variable comparative results for blood pressure outcomes require careful clinical interpretation. Background antihypertensive regimens were often intensified during follow-up, potentially masking true differences. These findings contextualize SGLT2 inhibitors within the broader landscape of post-transplant hypertension management. Current guideline-recommended antihypertensives each carry specific considerations in transplant recipients [15, 76, 77]. Calcium channel blockers may cause peripheral edema and interact with calcineurin inhibitor metabolism, while renin-angiotensin system inhibitors risk hyperkalemia and acute kidney injury, particularly problematic in patients with marginal allograft function [78, 79].

### Limitations and future directions

This study has several important limitations. First, this review involved a post-hoc adjustment in the original protocol to include single-arm (pre–post) analyses (a shift necessitated by the lack of comparative analysis).While this allowed for more comprehensive synthesis of the literature, changes observed over time within a single group cannot be interpreted as treatment effects, as they may reflect natural variation, regression to the mean, or changes in concurrent therapies rather than the intervention itself. These results should therefore be interpreted with caution and viewed as hypothesis-generating rather than definitive. The absence of systematic reporting for key safety endpoints such as genital infections, diabetic ketoacidosis, and acute kidney injury represents a major evidence gap. Additionally, inconsistent reporting and adjustment of concomitant antihypertensive therapies across studies introduce residual confounding that may have influenced blood pressure outcomes.

In addition, most included studies were observational and carried a serious risk of bias, which further limits causal interpretation. Confounding by indication remains a key concern [80]. Although the included randomized controlled trial was of high quality, it was relatively small and of short duration [34]. There was also considerable variability across studies in patient populations, SGLT2 inhibitor types, background treatments, and follow-up durations, which complicates interpretation. Finally, follow-up periods were relatively short, particularly for outcomes such as graft survival and long-term cardiovascular events, which may require several years to fully assess.

Furthermore, the majority of included studies evaluated SGLT2 inhibitors in the context of post-transplant diabetes or cardiorenal protection rather than hypertension treatment, and blood pressure outcomes were often secondary and variably measured. Changes in concomitant antihypertensive therapy were not consistently reported, introducing potential confounding. Our restriction to English-language articles may have introduced language bias, as relevant non-English studies could have been excluded. Although we searched clinical trial registries, unpublished or ongoing trials may not have been captured, potentially introducing publication bias.

These findings underscore the need for adequately powered randomized controlled trials in kidney transplant recipients with extended follow-up periods to capture effects on graft survival and cardiovascular events. Mechanistic sub-studies evaluating effects on calcineurin inhibitor pharmacokinetics, allograft histology, and arterial stiffness would enhance understanding.

## Conclusion

In controlled difference-in-differences analyses, SGLT2 inhibitors did not demonstrate statistically significant blood pressure reductions versus control in kidney transplant recipients. Exploratory single-arm analyses suggested modest within-group systolic blood pressure reductions and suggested metabolic effects, with an acceptable safety profile. However, most evidence derives from studies in which SGLT2 inhibitors were initiated for diabetes or cardiorenal protection, not for blood pressure control. Therefore, these findings characterize blood pressure effects rather than establish efficacy as primary antihypertensive therapy. Large randomized controlled trials with blood pressure as a primary endpoint are needed to determine their role in post-transplant hypertension management.

## Supplementary Information

Below is the link to the electronic supplementary material.

Supplementary file1 (DOCX 3360 KB)

## Data Availability

All data analyzed in this study were derived from published articles and publicly available sources. Extracted data used for the analyses are available from the corresponding author upon reasonable request.

## Abbreviations

CKD: Chronic kidney disease
ESRD: End-stage renal disease
KTR(s): Kidney transplant recipient(s)
SGLT2: Sodium–glucose cotransporter-2
SGLT2i: Sodium–glucose cotransporter-2 inhibitor(s)
BP: Blood pressure
SBP: Systolic blood pressure
DBP: Diastolic blood pressure
eGFR: Estimated glomerular filtration rate
HbA1c: Glycated hemoglobin
UTI: Urinary tract infection
RCT: Randomized controlled trial
PRISMA: Preferred Reporting Items for Systematic Reviews and Meta-Analyses
PROSPERO: International Prospective Register of Systematic Reviews
IRB: Institutional review board
RoB 2: Cochrane Risk of Bias Tool, Version 2
ROBINS-I: Risk of Bias in Non-Randomized Studies of Interventions
JBI: Joanna Briggs Institute
SD: Standard deviation
SE: Standard error
MDCFB: Mean difference of change from baseline
DiD: Difference in differences
GIV: Generic inverse variance
HK: Hartung–Knapp
I^2^: Higgins’ heterogeneity statistic
