# Supplementary material for: Blood pressure effects of SGLT2 inhibitors in kidney transplant recipients: a systematic review and meta-analysis
Source: Int Urol Nephrol. 2026 Apr 29;58(10):4303–18. doi: 10.1007/s11255-026-05162-9 (PMC13585813; doi:10.1007/s11255-026-05162-9)
Supplement: Supplementary file 1 [file 11255_2026_5162_MOESM1_ESM.docx]

**Table 1. Search Strategy for each database**

| **Database** | **Search String** |
| --- | --- |
| PubMed/Medline  (Results: 234) | ("Sodium-Glucose Transporter 2 Inhibitors"[Mesh] OR "sodium-glucose cotransporter 2 inhibitor*" OR "sodium-glucose transporter 2 inhibitor*" OR "SGLT2 inhibitor*" OR "SGLT-2 inhibitor*" OR empagliflozin OR dapagliflozin OR canagliflozin OR ertugliflozin OR ipragliflozin OR tofogliflozin OR luseogliflozin OR remogliflozin OR sotagliflozin OR gliflozin*) AND ("Kidney Transplantation"[Mesh] OR "Renal Transplantation"[Mesh] OR "kidney transplant*" OR "renal transplant*" OR "kidney allograft*" OR "renal graft*" OR "renal graft replacement" OR "kidney graft replacement" OR "renal allograft function" OR "kidney graft function" OR "graft function" OR "graft failure" OR "allograft failure" OR "renal allograft survival" OR "renal graft survival" OR "post-transplant" OR "post transplant" OR "post-transplantation" OR "post renal transplant" OR "post kidney transplant" OR "renal allograft recipient*" OR "kidney graft recipient*") AND ("Hypertension"[Mesh] OR "Blood Pressure"[Mesh] OR "Cardiovascular Diseases"[Mesh] OR "Heart Rate"[Mesh] OR "Pulse Pressure"[All Fields] OR hypertension OR "high blood pressure" OR "elevated blood pressure" OR "arterial hypertension" OR "post-transplant hypertension" OR "post transplantation hypertension" OR "systolic blood pressure" OR "diastolic blood pressure" OR "mean arterial pressure" OR "hemodynamic parameter*" OR "cardiovascular outcome*" OR "cardiovascular mortality" OR "major adverse cardiovascular event*" OR "MACE" OR "heart failure" OR "cardiac event*" OR "arterial stiffness" OR mortality OR "all-cause mortality" OR "overall survival" OR "patient survival" OR "Adverse Effects"[Mesh] OR "Adverse Event*"[All Fields] OR "Safety"[All Fields] OR "Treatment Outcome"[Mesh] OR "Complications"[Mesh] OR "Infection*"[Mesh] OR "urinary tract infection*" OR "UTI" OR "diabetic ketoacidosis" OR "DKA" OR "volume depletion" OR "dehydration" OR "acute kidney injury" OR "renal dysfunction" OR "graft rejection" OR "allograft rejection" OR "renal allograft rejection" OR "hospitalization" OR "Antihypertensive Agents"[Mesh] OR "antihypertensive therapy" OR "antihypertensive treatment" OR "blood pressure medication*" OR "standard antihypertensive" OR "placebo" OR "control group") |
| Scopus  (Results: 572) | (INDEXTERMS("sodium glucose transporter 2 inhibitor") OR TITLE-ABS-KEY("sodium-glucose cotransporter 2 inhibitor*" OR "sodium-glucose transporter 2 inhibitor*" OR "SGLT2 inhibitor*" OR "SGLT-2 inhibitor*" OR empagliflozin OR dapagliflozin OR canagliflozin OR ertugliflozin OR ipragliflozin OR tofogliflozin OR luseogliflozin OR remogliflozin OR sotagliflozin OR gliflozin*)) AND (INDEXTERMS("kidney transplantation") OR TITLE-ABS-KEY("kidney transplant*" OR "renal transplant*" OR "kidney allograft*" OR "renal graft*" OR "renal graft replacement" OR "kidney graft replacement" OR "renal allograft function" OR "kidney graft function" OR "graft function" OR "graft failure" OR "allograft failure" OR "renal allograft survival" OR "renal graft survival" OR "post-transplant" OR "post transplant" OR "post-transplantation" OR "post renal transplant" OR "post kidney transplant" OR "renal allograft recipient*" OR "kidney graft recipient*")) AND (INDEXTERMS("hypertension" OR "blood pressure" OR "cardiovascular disease" OR "heart rate" OR "adverse effect" OR "treatment outcome" OR "complication" OR "infection" OR "antihypertensive agent") OR TITLE-ABS-KEY("Pulse Pressure" OR hypertension OR "high blood pressure" OR "elevated blood pressure" OR "arterial hypertension" OR "post-transplant hypertension" OR "post transplantation hypertension" OR "systolic blood pressure" OR "diastolic blood pressure" OR "mean arterial pressure" OR "hemodynamic parameter*" OR "cardiovascular outcome*" OR "cardiovascular mortality" OR "major adverse cardiovascular event*" OR "MACE" OR "heart failure" OR "cardiac event*" OR "arterial stiffness" OR mortality OR "all-cause mortality" OR "overall survival" OR "patient survival" OR "Adverse Event*" OR "Safety" OR "urinary tract infection*" OR "UTI" OR "diabetic ketoacidosis" OR "DKA" OR "volume depletion" OR "dehydration" OR "acute kidney injury" OR "renal dysfunction" OR "graft rejection" OR "allograft rejection" OR "renal allograft rejection" OR "hospitalization" OR "antihypertensive therapy" OR "antihypertensive treatment" OR "blood pressure medication*" OR "standard antihypertensive" OR "placebo" OR "control group")) |
| Embase  (Results:606) | ('sodium glucose transporter 2 inhibitor'/exp OR 'sodium glucose cotransporter 2 inhibitor*':ab,ti,kw OR 'sodium glucose transporter 2 inhibitor*':ab,ti,kw OR 'SGLT2 inhibitor*':ab,ti,kw OR 'SGLT-2 inhibitor*':ab,ti,kw OR 'empagliflozin'/exp OR 'dapagliflozin'/exp OR 'canagliflozin'/exp OR 'ertugliflozin'/exp OR 'ipragliflozin'/exp OR 'tofogliflozin'/exp OR 'luseogliflozin'/exp OR 'remogliflozin':ab,ti,kw OR 'sotagliflozin'/exp OR 'gliflozin*':ab,ti,kw) AND ('kidney transplantation'/exp OR 'kidney transplant*':ab,ti,kw OR 'renal transplant*':ab,ti,kw OR 'kidney allograft*':ab,ti,kw OR 'renal graft*':ab,ti,kw OR 'renal graft replacement':ab,ti,kw OR 'kidney graft replacement':ab,ti,kw OR 'renal allograft function':ab,ti,kw OR 'kidney graft function':ab,ti,kw OR 'graft function':ab,ti,kw OR 'graft failure'/exp OR 'allograft failure':ab,ti,kw OR 'renal allograft survival':ab,ti,kw OR 'renal graft survival':ab,ti,kw OR 'post-transplant':ab,ti,kw OR 'post transplant':ab,ti,kw OR 'post-transplantation':ab,ti,kw OR 'post renal transplant':ab,ti,kw OR 'post kidney transplant':ab,ti,kw OR 'renal allograft recipient*':ab,ti,kw OR 'kidney graft recipient*':ab,ti,kw) AND ('hypertension'/exp OR 'blood pressure'/exp OR 'cardiovascular disease'/exp OR 'heart rate'/exp OR 'pulse pressure':ab,ti,kw OR 'hypertension':ab,ti,kw OR 'high blood pressure':ab,ti,kw OR 'elevated blood pressure':ab,ti,kw OR 'arterial hypertension':ab,ti,kw OR 'post-transplant hypertension':ab,ti,kw OR 'post transplantation hypertension':ab,ti,kw OR 'systolic blood pressure':ab,ti,kw OR 'diastolic blood pressure':ab,ti,kw OR 'mean arterial pressure':ab,ti,kw OR 'hemodynamic parameter*':ab,ti,kw OR 'cardiovascular outcome*':ab,ti,kw OR 'cardiovascular mortality'/exp OR 'major adverse cardiovascular event*':ab,ti,kw OR 'MACE':ab,ti,kw OR 'heart failure'/exp OR 'cardiac event*':ab,ti,kw OR 'arterial stiffness':ab,ti,kw OR 'mortality'/exp OR 'all-cause mortality':ab,ti,kw OR 'overall survival':ab,ti,kw OR 'patient survival':ab,ti,kw OR 'adverse drug reaction'/exp OR 'Adverse Event*':ab,ti,kw OR 'Safety':ab,ti,kw OR 'treatment outcome'/exp OR 'complication'/exp OR 'infection'/exp OR 'urinary tract infection'/exp OR 'UTI':ab,ti,kw OR 'diabetic ketoacidosis'/exp OR 'DKA':ab,ti,kw OR 'volume depletion':ab,ti,kw OR 'dehydration'/exp OR 'acute kidney injury'/exp OR 'renal dysfunction':ab,ti,kw OR 'graft rejection'/exp OR 'allograft rejection':ab,ti,kw OR 'renal allograft rejection':ab,ti,kw OR 'hospitalization'/exp OR 'antihypertensive agent'/exp OR 'antihypertensive therapy':ab,ti,kw OR 'antihypertensive treatment':ab,ti,kw OR 'blood pressure medication*':ab,ti,kw OR 'standard antihypertensive':ab,ti,kw OR 'placebo'/exp OR 'control group':ab,ti,kw) |

**Table 2. Baseline Characteristics of Included Comparative Studies**

| Study ID | Age,  Mean ±SD, years | | Male (n%) | | SBP, mmHG  Mean±SD | | Mean DBP,  MmHG  Mean±SD | | Body weight, kg  Mean ±SD | | eGFR, mL/min per 1.73 m2,  Mean ±SD | | HbA1c, %  Mean ±SD | | Creatinine,mg/dl, Mean ±SD | |
| --- | --- | --- | --- | --- | --- | --- | --- | --- | --- | --- | --- | --- | --- | --- | --- | --- |
|  | SGLT2i | Control | SGLT2i | Control | SGLT2i | Control | SGLT2i | Control | SGLT2i | Control | SGLT2i | Control | SGLT2i | Control | SGLT2i | Control |
| Halden 2019 (31) | 63  (31,72)* | 59(21,75)* | 77 | 77 | 143  (111,176)* | 140  (100,163)* | 79  (63,94)* | 82  (55,94)* | 92.0  (81.8,104.5) | 84.0  (79.3,94.0)* | 66  (41,83)* | 59  (44,82)* | 6.9  (6.5,8.2) * | 6.8  (6.1,7.2)* | NR | NR |
| Hisadome 2021  (35) | 54.8  ± 7.0 | 55.7 ± 9.5 | 79 | 74 | 123 ± 16 | 130 ± 15 | 74 ± 10 | 73 ± 10 | 26.1 ± 4.0 | 25.5 ± 3.4 | 50.4 ± 13.9 | 47.5 ± 13.1 | 7.7 ± 0.9 | 7.6 ± 1.3 | NR | NR |
| Demir 2023 (38) | 49.1± 11.2 | 55.0 ± 9.7 | 66 | 56 | NR | NR | NR | NR | NR | NR | 71.9 ± 18.2 | 73.2 ± 19.7 | 9.1 ±  2.2 | 6.4 ±  1.0 | 1.06 ±  0.26 | 1.14 ±  0.29 |
| Yeggalam 2023  (36) | 61.7 ± 11.0 | 61.4±  9.7 | 66 | 61 | 132.8 ±  20.6 | 138.1 ±  17.0 | 73.4 ±  11.1 | 74.9± 11.4 | 95.8± 15.6 | 93.8 ± 20.6 | 55.9 ± 18.6 | 52.6 ± 19.3 | 7.9 ± 1.8 | 7.3 ± 1.5 | 1.4 ± 0.4 | 1.5 ± 0.6 |
| Mahmoud 2023  (37) | 56 | 57 | 67 | 73 | 137 | 128 | 78 | 76 | NR | NR | 67.2 | 63.9 | 8.0 | 7.2 | NR | NR |
| *These studies reported Median with IQR for the highlighted value  NR= Did not report the value.  S SGTL2i: Sodium-glucose cotransporter-2 inhibitors | | | | | | | | | | | | | | | | |

**Table 3. Baseline Characteristics of Included Single Arm Studies**

| Study ID | Age,  Mean ± SD, years | Male (n%) | SBP, mmHG  Mean ± SD | Mean DBP,  MmHG  Mean ± SD | Body weight, kg  Mean ± SD | eGFR, mL/min per 1.73 m2,  Mean ± SD | HbA1c, %  Mean ± SD | Creatinine,mg/dl, Mean ± SD |
| --- | --- | --- | --- | --- | --- | --- | --- | --- |
| Bilancio 2025  (42) | 55.1 (41.8–62.1)* | 82.8 | 139 ± 15 | 83 ± 10 | 81 ± 16 | 56 ± 18 | NR | 1.47 ± 0.45 |
| Maigret 2024  (40) | 62.6[52.1–69.5]* | 76.4 | 143[132–155]* | 81[73–89]* | 82.0[72.0–91.4]* | 44.8[37.1–54.5]* | NR | 142[113–173]* |
| Fructoso 2023  (39) | 61.6±9.9 | 73.7 | 137(135–139)* | 75.8(74.5–77.1)* | 81.5(79.4–83.6)* | 58.4(56.2–60.6)* | 7.56(7.41–7.71)* | NR |
| Shah 2019  (34) | 53.8±7.1 | 98 | 142 ± 21 | 81 ± 9 | 78.6 ± 12.1 | NR | 8.5 ± 1.5 | 1.1 ± 0.2 |
| Schwaiger 2018  (32) | 56.5 ±7.9 | 50 | 150.0 ± 25.6 | 86.1 ± 14.0 | 74.8 ± 17.2 | 55.6 ± 20.3 | 6.5 ± 0.8 | 1.3 ± 0.4 |
| Quilis 2024  (41) | 58(52-66)* | 68.2 | 130(122-140)* | 80(70-80)* | 77.6(70-86.5)* | 31(23.7-45.2)* | NR | 2.1(1.48-2.85)* |
| AlKindi 2019  (33) | 56.8 ± 13.70 | 75 | 135 ± 9.59 | 80.63 ±  10.13 |  | 75.75 ±  13.38 | 9.34 ± 1.36 | 92.511.65 |
| *These studies reported Median with IQR for the highlighted value  NR= Did not report the value. | | | | | | | | |

**Table 4. Joanna Briggs Institute (JBI) Critical Appraisal Checklist for Case Series for Single Arm Studies**

| JBI Criterion | Shah et al. (2019) | Bilancio et al. (2025) | Maigret et al. (2025) | Sánchez Fructuoso et al. (2023) | Schwaiger et al. (2019) | Quilis et al. (2025) | AlKindi et al. (2020) |
| --- | --- | --- | --- | --- | --- | --- | --- |
| 1. Were there clear criteria for inclusion in the case series? | Yes | Yes | Yes | Yes | Yes | Yes | Yes |
| 2. Was the condition measured in a standard, reliable way for all participants included in the case series? | Yes | Yes | Yes | Yes | Yes | Yes | Yes |
| 3. Were valid methods used for identification of the condition for all participants included in the case series? | Yes | Yes | Yes | Yes | Yes | Yes | Yes |
| 4.Did the case series have consecutive inclusion of participants? | No | Unsure | Yes | Yes | No | Yes | Yes |
| 5. Did the case series have complete inclusion of participants? | No | Unsure | No | No | No | Yes | Yes |
| 6. Was there clear reporting of the demographics of the participants in the study? | Yes | Yes | Yes | Yes | Yes | Yes | Yes |
| 7. • Was there clear reporting of clinical information of the participants? | Yes | Yes | Yes | Yes | Yes | Yes | Yes |
| 8. Were the outcomes or follow up results of cases clearly reported? | Yes | Yes | Yes | Yes | Yes | Yes | Yes |
| 9. Was there clear reporting of the presenting site(s)/clinic(s) demographic information? | Yes | Yes | Yes | Yes | Yes | Yes | Yes |
| 10. Was statistical analysis appropriate? | No | Yes | Yes | Yes | Yes | Yes | No |

**Table 5. GRADE Assessment Table:**

| Test Important Outcome | Time point | Effects | No. of Studies | Quality of Evidence | Comment | Certainty Domains Affected |
| --- | --- | --- | --- | --- | --- | --- |
| SBP | 3 months | −5.66 (−6.86 to −4.45) | 4 | LOW | Downgraded for observational studies (mostly single arm designs) with serious risk of bias. | Risk of bias |
|  | 6 months | −4.80 (−7.24 to −2.37) | 9 | LOW | Downgraded for observational study designs with serious risk of bias. | Risk of bias |
|  | 12 months | −2.73 (−9.99 to +4.53) | 5 | VERY LOW | Downgraded for observational study designs with serious risk of bias, substantial heterogeneity (I^2^ = 76.4%) and imprecision. | Risk of bias, inconsistency, imprecision |
| Body weight | 12 months | -4.90 (-9.59 to -0.21) | 2 | LOW | Downgraded for observational study designs with serious risk of bias and imprecision (wide confidence interval). | Risk of bias, imprecision |
| UTI | - | +0.55 (+0.28 to +1.09) | 4 | LOW | Downgraded for retrospective observational study designs with serious risk of bias and imprecision. | Risk of bias, imprecision |
| eGFR | 3 months | Not pooled due to insufficient number of studies reporting comparable data | 2 | VERY LOW | Downgraded for retrospective observational study designs with serious risk of bias and imprecision. | Risk of bias, imprecision |
|  | 6 months | Not pooled due to insufficient number of studies reporting comparable data | 2 | VERY LOW | Downgraded for retrospective observational study designs with serious risk of bias and imprecision. | Risk of bias, imprecision |
|  | 12 months | 4.30 (−0.32 to 8.93) | 3 | VERY LOW | Downgraded for retrospective observational study designs with serious risk of bias, inconsistency ( I^2^ = 68%), and imprecision. | Risk of bias, inconsistency, imprecision |

**Table 6. Sensitivity Analysis of Pooled Mean Differences Across Variable Correlation Coefficients**

| Outcome | Timepoint | k | Corr = 0.3 (MD [95% CI]) | Corr = 0.5 (Primary) | Corr = 0.7 (MD [95% CI]) |
| --- | --- | --- | --- | --- | --- |
| SBP | 3 Months | 4 | -5.66 [-6.88, -4.44] | -5.66 [-6.86, -4.45] | -5.65 [-6.82, -4.47] |
|  | 6 Months | 9 | -4.65 [-6.62, -2.68] | -4.80 [-7.24, -2.37] | -5.06 [-7.91, -2.21] |
|  | 12 Months | 5 | -2.65 [-9.97, +4.67] | -2.73 [-9.99, +4.53] | -2.84 [-9.99, +4.31] |
| DBP | 3 Months | 3 | -2.67 [-6.32, +0.99] | -2.67 [-6.31, +0.96] | -2.68 [-6.27, +0.91] |
|  | 6 Months* | 7 | -3.30 [-6.21, -0.40] | -3.41 [-6.59, -0.23] | -3.57 [-7.09, -0.04] |
|  | 12 Months | 5 | -2.85 [-6.32, +0.63] | -3.11 [-7.17, +0.95] | -3.34 [-7.86, +1.18] |
| Footnote: Abbreviations: CI, confidence interval; Corr, correlation coefficient; DBP, diastolic blood pressure; k, number of studies; MD, mean difference; SBP, systolic blood pressure.  * For the 6-month DBP analysis, results are presented following the exclusion of AlKindi et al. (36), identified as a clinically implausible outlier and suspected typographical error in the primary source. | | | | | |

**Table 7. Subgroup analyses for SBP and DBP at 3, 6, and 12 months.**

| **Outcome** | **Subgroup Category** | **Factor** | **k** | **MD** | **95% CI** | **I2 (%)** | **p-value (Between)** |
| --- | --- | --- | --- | --- | --- | --- | --- |
| **SBP 6-Month** | **DM Status** | Mixed | 5 | -4.94 | [-7.62, -2.26] | 22.7% | **0.667** |
|  |  | Diabetic | 3 | -6.54 | [-26.51, 13.43] | 64.3% |  |
|  |  | Non-Diabetic | 1 | -2.60 | [-7.94, 2.74] | -- |  |
|  | **Med Status** | Stable | 3 | -3.38 | [-9.43, 2.66] | 0.0% | **0.219** |
|  |  | Adjusted | 6 | -5.89 | [-9.70, -2.09] | 41.2% |  |
|  | **Tx Period** | Late (> 1yr) | 7 | -4.57 | [-7.72, -1.42] | 42.0% | **0.017*** |
|  |  | Mixed/Early | 2 | -7.75 | [-12.00, -3.50] | 0.0% |  |
|  | **HTN Status** | Normal | 5 | -5.10 | [-7.47, -2.73] | 1.1% | **0.933** |
|  |  | High | 4 | -4.86 | [-13.65, 3.94] | 53.7% |  |
|  | **BP Method** | Office | 2 | -3.10 | [-34.92, 28.72] | 45.2% | **0.431** |
|  |  | Routine | 7 | -5.31 | [-8.45, -2.18] | 34.8% |  |
| --- | --- | --- | --- | --- | --- | --- | --- |
| **DBP 6-Month** | **DM Status** | Mixed | 4 | -2.99 | [-4.96, -1.02] | 34.4% | **0.566** |
| (Outlier Excl.) |  | Diabetic | 3 | -5.60 | [-25.00, 13.80] | 85.7% |  |
|  | **Med Status** | Stable | 2 | -2.96 | [-32.00, 26.08] | 75.4% | **0.828** |
|  |  | Adjusted | 5 | -3.59 | [-8.60, 1.42] | 71.4% |  |
|  | **Tx Period** | Late (>1yr) | 5 | -3.86 | [-9.02, 1.29] | 78.4% | **0.344** |
|  |  | Mixed/Early | 2 | -2.10 | [-4.29, 0.09] | 0.0% |  |
| --- | --- | --- | --- | --- | --- | --- | --- |
| **SBP 3-Month** | **Med Status** | Stable | 1 | -6.00 | [-9.46, -2.54] | -- | **0.793** |
|  |  | Adjusted | 3 | -5.52 | [-7.75, -3.29] | 0.0% |  |
|  | **Tx Period** | Late (\ge1yr) | 3 | -5.70 | [-7.52, -3.88] | 0.0% | **0.760** |
|  |  | Mixed/Early | 1 | -3.90 | [-15.42, 7.62] | -- |  |
| --- | --- | --- | --- | --- | --- | --- | --- |
| **DBP 3-Month** | **DM Status** | Mixed | 2 | -3.03 | [-4.05, -2.01] | 0.0% | **0.246** |
|  |  | Diabetic | 1 | 1.30 | [-6.02, 8.62] | -- |  |
|  | **Med Status** | Stable | 1 | -3.00 | [-5.34, -0.66] | -- | **0.496** |
|  |  | Adjusted | 2 | -1.26 | [-29.94, 27.41] | 0.0% |  |
| --- | --- | --- | --- | --- | --- | --- | --- |
| **SBP 12-Month** | **Med Status** | Adjusted | 4 | -5.14 | [-6.82, -3.46] | 0.0% | **<0.0001*** |
|  |  | Stable | 1 | 6.00 | [0.81, 11.19] | -- |  |
|  | **DM Status** | Mixed | 2 | -5.21 | [-15.91, 5.49] | 0.0% | **0.079** |
|  |  | Diabetic | 3 | 1.10 | [-13.95, 16.16] | 49.1% |  |
| --- | --- | --- | --- | --- | --- | --- | --- |
| **DBP 12-Month** | **HTN Status** | Normal | 4 | -2.21 | [-3.93, -0.49] | 0.0% | **0.024*** |
|  |  | High | 1 | -10.00 | [-16.69, -3.31] | -- |  |
|  | **Med Status** | Adjusted | 4 | -3.81 | [-10.51, 2.89] | 58.0% | **0.523** |
|  |  | Stable | 1 | -2.00 | [-5.70, 1.70] | -- |  |
| Footnote: Rationale for Subgroup Exclusions and Heterogeneity Reporting   - Heterogeneity (I^2^): Values represent the percentage of total variation across studies that is due to heterogeneity rather than chance. For subgroups consisting of a single study (k=1), I^2^ cannot be mathematically determined and is indicated by a dash (--). - Rationale for Category Exclusion: Subgroup analyses were only performed where at least two distinct clinical categories were present among the studies included for a specific outcome.   - SBP 3-Month: DM Status was excluded as all four studies represented Mixed or Diabetic cohorts with insufficient distribution for a three-way comparison.   - DBP 3-Month: HTN Status was excluded because all included studies represented cohorts with Normal baseline SBP (<140 mmHg).   - SBP/DBP 12-Month: BP Method and Tx Period were excluded as all five studies utilized Routine Clinic measurements and primarily represented Late post-transplant cohorts (>1 year), precluding between-group statistical contrast. - Significance: Asterisks (*) denote statistical significance at p < 0.05 based on the between-group test.   **Abbreviations:** **BP:** Blood Pressure; **CI:** Confidence Interval; **DM:** Diabetes Mellitus; **HK:** Hartung-Knapp adjustment; **HTN:** Hypertension (Baseline status: High >140 mmHg SBP; Normal < 140 mmHg SBP); **I^2^:** Heterogeneity statistic; **k:** Number of studies; **MD:** Mean Difference (mmHg); **Med Status:** Medication stability status (Stable vs. Adjusted antihypertensive regimen); **SBP/DBP:** Systolic/Diastolic Blood Pressure; **Tx Period:** Post-transplant period (Late >1 year; Mixed/Early < 1 year). | | | | | | | |

**Figure S1. Risk of Bias of Individual studies**

**Figure S2. Risk of Bias results for individual domains**

**Figure S3**. **Forest plot of change from baseline in systolic blood pressure at 3 months**

**Figure S4. Forest plot of change from baseline in systolic blood pressure at 6 months**

**Figure S5. Forest plot of change from baseline in systolic blood pressure at 12 months**

**Figure S6. Forest plot of change from baseline in diastolic blood pressure at 3 months**

**Figure S7. Forest plot of change from baseline in diastolic blood pressure at 6 months**

**Figure S8. Change from baseline in diastolic blood pressure at 6 months excluding AlKindi et al.**

**Figure S9. Forest plot of change from baseline in diastolic blood pressure at 12 months**

**Figure S10. Forest plot of change from baseline in HbA1c at 6 months**

**Figure S11. Forest plot of change from baseline in body weight at 12 months**

**Figure S12. Forest plot of change from baseline in estimated glomerular filtration rate at 6 months**

**Figure S13. Forest plot of change from baseline in serum creatinine at 12 months**

**Figure S14. Forest plot of change from baseline in urine protein-to-creatinine ratio at 12 months**

**Figure S15. Forest plot of additional sensitivity analysis forest plot**

**Risk of Bias Report**

Halden et al., the only randomized controlled trial included in this review, was judged to have a low risk of bias across all domains using the Cochrane Risk of Bias 2 (RoB 2) tool.

Using the ROBINS-I tool, all 11 observational studies were assessed as having a serious risk of bias (**Supplemental Figures 1 and 2**). Nine studies exhibited serious risk in Domain 1 (bias due to confounding). All single-arm studies lacked a comparator group to account for regression to the mean or natural disease progression, while two comparative observational studies (Demir et al. and Mahmoud et al.) did not adequately adjust for baseline differences between groups.

Serious risk was also identified in Domain 2 (bias in selection of participants into the study) and Domain 5 (bias due to missing data). All four comparative studies excluded patients who discontinued SGLT2 inhibitor therapy before a predefined minimum duration, raising the possibility that early treatment failures were excluded and resulting in a survivor-type cohort. Additionally, four single-arm studies applied strict exclusion criteria that preferentially selected clinically stable patients with favorable prognostic profiles.

Bias due to deviations from intended interventions (Domain 4) was rated as moderate in nearly half of the observational studies. This was primarily related to changes in concomitant therapies—such as insulin dose reduction or antihypertensive adjustment—during follow-up, as well as insufficient reporting of treatment adherence, both of which may have confounded the observed effects of SGLT2 inhibitors. Furthermore, five studies were assigned a moderate risk in Domain 7 (bias in selection of the reported result) due to the absence of a pre-registered protocol, precluding exclusion of selective outcome reporting.

The Joanna Briggs Institute (JBI) Critical Appraisal Checklist for Case Series was used to assess the internal validity of the seven single-arm studies (**Supplementary Table 4**). Three studies were rated as high quality. Schwaiger et al. was rated as moderate quality due to substantial attrition and restrictive eligibility criteria. The remaining studies were judged to be of low quality because of small sample sizes and limited statistical power.
